# The PubChem chemical structure sketcher

**DOI:** 10.1186/1758-2946-1-20

**Published:** 2009-12-17

**Authors:** Wolf D Ihlenfeldt, Evan E Bolton, Stephen H Bryant

**Affiliations:** 1 Xemistry GmbH, Hainholzweg 11, D-61462 Königstein, Germany; 2 National Center for Biotechnology Information, National Library of Medicine, National Institutes of Health, Department of Health and Human Services, 8600 Rockville Pike, Bethesda, MD 20894, USA

## Abstract

PubChem is an important public, Web-based information source for chemical and bioactivity information. In order to provide convenient structure search methods on compounds stored in this database, one mandatory component is a Web-based drawing tool for interactive sketching of chemical query structures. Web-enabled chemical structure sketchers are not new, being in existence for years; however, solutions available rely on complex technology like Java applets or platform-dependent plug-ins. Due to general policy and support incident rate considerations, Java-based or platform-specific sketchers cannot be deployed as a part of public NCBI Web services. Our solution: a chemical structure sketching tool based exclusively on CGI server processing, client-side JavaScript functions, and image sequence streaming. The PubChem structure editor does not require the presence of any specific runtime support libraries or browser configurations on the client. It is completely platform-independent and verified to work on all major Web browsers, including older ones without support for Web2.0 JavaScript objects.

## Introduction

The National Center for Biotechnology Information (NCBI) http://www.ncbi.nlm.nih.gov is widely known for its cluster of literature, biology, genomics, and chemistry databases which are freely accessible on the Web [1]. These databases register many tens of millions of hits per day from millions of users accessing the site via a broad range of Web browsers and operating system platforms. From experience gained in more than a decade of operation, NCBI has developed a set of principles concerning what kind of client-side technology can be deployed as a part of its Web services. These help to prevent access or support issues, specifically those pertaining to configuration or installation of the user's Internet access tools. Therefore, any technology which cannot be expected to work reliably with a standard client browser installation right out of the box, typically cannot be used in public NCBI Web services. For these reasons, NCBI Web pages traditionally allow only the use of basic HTML and JavaScript on the client, CGI/FCGI processes on the server, and nothing more.

This approach has not been a severe limitation for the operation of the classic collection of databases served by NCBI. However, with the addition of the PubChem[2] suite of databases, an obvious problem arose. Chemical structure databases cannot be readily queried by structure in a reasonable fashion using purely textual input and standard HTML form elements. Users of chemical structure databases demand the capability to search by full-structure or substructure, with a graphical rendition of the query structure as input. Fragment name search, externally generated and pasted SMILES/SMARTS[3]strings, or upload of query files drawn with stand-alone chemical structure drawing programs are awkward procedures and do not yield a satisfactory user experience. It became very clear that the PubChem structure search system needed a method to allow users to draw their query structure interactively, in an integrated fashion, and without the need of external software.

This is, of course, not a new problem. Most of the structure databases on the Web already have graphical structure input tools. These traditionally come in two styles: Java applets and browser plug-ins. A Java applet requires that the client browser has a functional Java virtual machine installed and correctly configured. While this may be a condition easily satisfied by most browsers, there will always be some fraction where an issue may crop up. For example, if only one in ten thousand of NCBI's two million world-wide users per day encounters and reports such an issue, two hundred user requests will ensue, distracting and diluting support capabilities. So, while there are quite a number of capable Java-based structure editors such as JME[4], JChemPaint[5]http://sourceforge.net/projects/jchempaint/, JmolDraw[6], MCDL[7], SDA[8], or Marvin[9], the use of any of these for PubChem was not possible. Relying on plug-ins such as the one from ChemDraw[10], is even more problematic because plug-ins are platform-dependent, and there are (to our knowledge) no free chemical structure editor plug-ins which can connect to arbitrary sites.

A very recent trend are first attempts to implement chemical structure sketchers purely based on advanced client-side JavaScript functionality, without required server interaction. One example for this approach is *jsmoleditor*[11]. Disadvantages of this approach are that these applications only work on very recent browser releases and make necessary limitations in the feature set. For example, 2D-layout cleanup and import/export of various chemical file formats typically involve a rather large code base to implement them. Sending a large amount of JavaScript code to a browser client in a dynamic per-session fashion is not currently feasible. Nevertheless, it is not unlikely that future Web sketchers will use more and more JavaScript client-side intelligence and rely on server-side functions only for more demanding computational tasks.

## Design Considerations

Facing restrictions on the use of Java or platform-specific solutions, we decided to go a completely new route in implementing a full featured, dynamically interactive Web-based structure editor. Like the approach pioneered by in internal CIBA project[12] and then also taken up, according to one of the reviewers as a direct result of seeing the CIBA prototype, by Daylight Grins http://www.daylight.com/daycgi/testgrins, we considered to move all the chemical structure processing functions of a structure editor onto a server CGI, and to send a dynamically generated sequence of images with the evolving structure back to the client browser, but, unlike Daylight Grins, the editor behaves much like an interactive application with mouse movements being tracked as they are being made, rather than requiring the user click on an image to register their action. This type of software configuration would not require any applets or plug-ins on the client and appeared to be feasible on any browser supporting the display of images and basic JavaScript functions

The WebME editor from Molinspiration, with a design very similar to the PubChem sketcher, was released after the PubChem sketcher had been publicly deployed for several months. At the time of writing, it re-used functions of the portable JavaScript mouse event code originally written by us for the PubChem Sketcher. This code re-use is not problematic - the sketcher JavaScript components are US Government Work in the public domain. We have been informed that the current version of this software is now relying on a different event catching mechanism.

The bandwidth requirements for this model are not excessive. Essentially, the client needs to capture and send mouse events on an image area (not more than a couple of dozen bytes per second), and to receive a sequence of images. Since typical chemical structure drawings consist mostly of a monochrome background, with sparse monochrome lines and letters, these images were expected to compress very well. They do not exceed a couple of kilobytes for a reasonable-sized drawing area. Even with four or five image updates per second, the required receiver bandwidth would thus not exceed some ten kilobytes per second. This poses certainly no problem for Internet access via broadband and could be, after some throttling of the data stream, acceptable even for dial-up via traditional phone line or ISDN connections. It is comparable to the bandwidth requirements of Internet radio or telephony, and certainly far less than streaming video. A segmentation of the drawing area into panels in order to further reduce the amount of data sent to the client was considered, but not deemed necessary after these initial calculations.

The server responsible for the update of the images must be able to process multiple events per second in order to guarantee a satisfactory user experience. Users expect dynamic feedback as it is provided in standard stand-alone structure editors, such as continuously updated bond lines following the mouse cursor. Because of the rapid sequence of events, the server CGI application has to be of the FastCGI (FCGI)[13] variant, as even the 100 ms start-up overhead of a classical CGI to process only an individual event would be prohibitive.

The general rules for the deployment of Web applications at NCBI also require robustness and redundancy with fail-over support. The sketcher system as it is currently deployed at PubChem uses two independent multi-processor server hosts, and redundant database servers for storing state. To the user, this is invisible - servers are transparently switched depending on the load during a drawing session, and multiple server processes are running in parallel on each host. Nevertheless, these requirements for redundancy and parallelism needed to be taken into account early in the design and coding of the application because they are difficult to retrofit at a later stage.

## Core Technology

The PubChem sketcher system is at its core a comparatively simple CACTVS[14]http://www.xemistry.com cheminformatics toolkit application script. All sketcher functionality on the server is implemented in ~2100 lines of script code. Such efficiency is achieved considering CACTVS provides most features required to code a structure editor. This includes: basic handling of chemical structure objects; creation, deletion and modification of atoms and bonds; a structure layout/cleanup algorithm; I/O modules for the input and output of many structure exchange formats, such as MDL MOL/SDF, ChemDraw CDX/CDXML, ISIS drawing formats, ACD/Labs ChemSketch files, Daylight SMILES/SMARTS, and InChI; basic image output functionality, although some extra functionality was added for this purpose (detailed in the next section); FCGI and CGI data input and decoding functions; and HTTP header output function, in its default configuration. With such capabilities, writing a server for this task is not difficult. Because the CACTVS script commands are high-level, their execution as interpreted statements is not a bottleneck. More than 90% of the execution time spent in response to a client request is spent in C-coded library routines and not the script interpreter.

As part of the general integration of CACTVS for the use within the PubChem project, we added a couple of general-purpose interface functions to the toolkit. These include encoding and decoding of the PubChem ASN.1 binary and ASCII chemical structure representations and functions to access the PubChem structure database and queuing system. These are used in the specific version of the sketcher deployed at NCBI in order to integrate the application into the PubChem environment. However, if no site-specific functions, such as state storage in the queuing system or non-public direct retrieval of PubChem structures via compound (CID) or substance (SID) identifiers, are needed, the PubChem sketcher server process can be run with a standard interpreter from the CACTVS toolkit.

## Image generation

One of the most critical functions of the sketcher is the image generation functionality. At the lowest level, we are using the GD library[15] to render the structure images, sometimes with additional graphical feedback objects such as lines and rectangles. Images are formatted in memory as binary blobs and transferred to the client. The default image format sent to the client browser is PNG[16]. {Browsers not supporting PNG, e.g., Microsoft Internet Explorer (IE) 4.0, are detected by JavaScript and default to GIF[17] image format.} The reason for this choice is that PNG formatted images turned out to be smaller than GIF formatted images after standard processing. Table 1 shows the image size for PubChem Compound CID 2244 (aspirin) (http://pubchem.ncbi.nlm.nih.gov/summary/summary.cgi?cid=2244) drawn in the style used in the sketcher (550 × 380 pixels, black atom symbols and bond lines, white background, not interlaced).

**Table 1:** Image sizes for PubChem Compound CID 2244 as a function of image format.

*Format*	*Anti-aliased Drawing*	*Byte count*
24-bit true color PNG	yes	8 511
8-bit colormap GIF, dithered	yes	3 778
8-bit colormap GIF, color reduced	yes	4 689
8-bit colormap PNG, color reduced	yes	4 254
8-bit colormap GIF, original colormap	no	2 151
8-bit colormap PNG, original colormap	no	1 733

By default, we draw both element symbols and lines in anti-aliased fashion to improve visual appearance. Internally, this requires rendering on a 24-bit image object because anti-aliasing requires many (often hundreds) intermediate color shades. However, original true-color 24-bit images are big - too big to be readily sent directly to the client. Therefore, we reduce the color space in a postprocessing step and transmit only 8-bit colormap-indexed images. The GD library natively provides a colorspace reduction function based on dithering. However, this class of algorithm is primarily suited to photographs and other images with large colored regions with comparatively subtle color changes from one pixel to the next. Line drawings deteriorate notably and become fuzzy when processed in this manner. In order to address this problem, we wrote a custom colorspace reduction function which simply determines the most common 24-bit colors, with additional weight for colors far from any color already represented as a colormap entry. All 24-bit colors in the original image are then rounded to the closest colormap entry. This resulting reduced 8-bit colormap image is used for display. Each processed image varies in size but is typically about 4 Kbytes. This size range is unfortunately prone to encounter a size-related IE browser PNG rendering bug http://support.microsoft.com/?kbid=822071. We pad images in invisible comment fields that have no effect on the display, as required to circumvent this problem

With an update rate of four images per second, and the HTTP header and TCP protocol overhead not exceeding a few percent, the result is a bandwidth requirement of roughly 128 Kbit/s. This is about the same as a quality MP3 radio stream. Nevertheless, this may still be too much for dialup connections or users experiencing significant network congestion to the PubChem website. In order to accommodate users lacking sufficient network bandwidth, a network speed control element to the user interface is available. If the low-bandwidth option is chosen, anti-aliasing is disabled (resulting in images with a somewhat less pleasing visual appearance but that are less than half the size of the smoothed version) and thus the required sketcher bandwidth is within the capacity of a single-channel ISDN connection (64 Kbit/s), even without the reduction of the processed event rate (see below) which is simultaneously enforced. These "low-speed" images are directly rendered as 8-bit colormap images. Because the sketcher uses only a few primary drawing colors (black, red, blue, green, orange), there is no danger of colormap overflow when not using anti-aliasing.

Under special circumstances, we send an additional type of image to the client. In order to provide easily recognizable visual feedback for error conditions, parts of the structure (e.g., the offending atom, bond, or fragment) or the whole image area are flashed orange. In order to deliver a clear signal with reliable timing, and in order to avoid the complication of having to send error condition flags back to the client which then would need to request additional images in the highlight sequence, we assemble a non-cycling animated GIF in and transmit it as a single image. If the flash area is small, this animated GIF is not significantly different in size from a standard static image, because the animated GIF specification allows the time-controlled replacement of arbitrary sections of the base image by small bitmaps. The last operation on the flash sequence is to restore the original drawing, so that the image can be retained as drawing area backdrop for normal operation. An animated variant of the PNG format has been defined[18], but at this time it is not sufficiently supported on Web browsers. The GD library (at the time of writing) does not support the assembly of animated GIF images. We had to add this functionality.

## Client Side Processing

**Figure 1:**
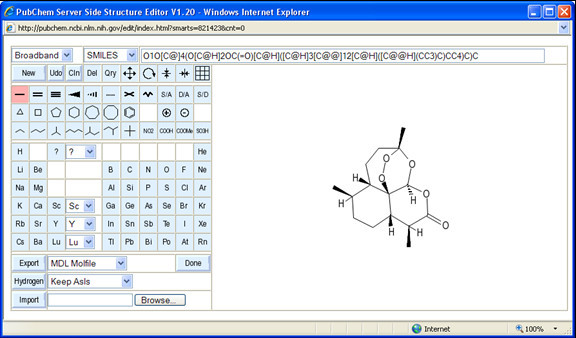
**The PubChem sketcher main window, with loaded compound**. The sketcher contains three visible sub-window sections: a drawing area (right), a mode control pad (left), and a status line (top).

The part of the PubChem sketcher which is loaded by the client browser consists of a couple of simple HTML pages with embedded JavaScript functions. The main window (see Figure 1) has three visible components and one invisible component. The right section is the drawing area. This area is (on recent browsers) covered by a simple 8-bit colormap PNG image with the current structure. In response to mouse events, it is dynamically replaced by new server-generated images. Image replacements are requested by client JavaScript functions - this is not a continuous image stream. The left section of the main window controls the sketcher mode. Besides a grid of buttons and pull-down menus, whose visual appearance is toggled by JavaScript utility functions and mirrors the internal state of the sketcher, this area also contains (in its underlying HTML encoding) a Web form used to transmit certain information to/from the client, for example to request import and export operations. The top section of the main window is the status line, which displays continuously updated textual information about the current structure. Depending on the display style settings, this field can show: SMILES, SMART, InChI, or SLN strings; or molecular formula (provided with molecular weight). This display area is not just a passive display - it is also possible to paste string representations of chemical structures into this field and add them to the current structure by hitting the return key.

A final component to the main window is a hidden frame, which is not visible but essential for operation. This frame is, besides the drawing area image, a second target of server output. After a request by a client JavaScript function, the server will send synthesized JavaScript commands into this frame, which (upon execution) update the status line or transfer the current structure in various encodings to Web pages that are linked to the sketcher and want to receive the resulting structure data (e.g., the input form of a structure search page). In order to facilitate data exchange with other Web pages via cross-window scripting, all sketcher pages operate in a configurable JavaScript document domain, usually set to a less specific domain than that of the fully qualified host the sketcher pages are served.

The most complicated task of the client-side JavaScript code is to capture mouse events on the drawing area. These events are then filtered, and at appropriate times an update request is made to update the drawing area images and/or the status line. The image is requested from the server via a simple CGI-style GET URL, with parameters encoding the current sketcher mode and event details. Since there can be an arbitrary number of parallel editing sessions, and the PubChem server set-up employs multiple hosts and parallel server processes, it is necessary to identify each session. This is conveniently achieved by a pseudo-random session ID that is automatically generated by a client-side JavaScript function the first time the main window page is loaded. The session ID is, from then on, a part of all future CGI URL parameters for that sketcher session. Besides generating a session ID, the start-up code also supports the pre-loading of the sketcher with structure data from various sources, for example, SMILES or SMARTS strings or PubChem CID, SID, or PubChem Deposition System identifiers (used to edit structural data in the process of being added to PubChem). This is done by decoding the initial startup URL of the sketcher main window and sending properly formatted preload instructions to the server when requesting the first drawing area image.

Event management on the client consists of two main tasks. First, event handlers for all events of interest need to be registered. In a second step, these events must be filtered and reduced to a manageable number and frequency. Catching events is (unfortunately) far from standard between different browsers. As a relic from the "browser wars" of the 1990's, there are a number of mutually incompatible event models in different Web browsers. Fortunately, tutorials and sample code on how to capture and interpret events on all major browsers are not difficult to find on the Web. Interested readers may want to inspect the source code of the main sketcher window. All involved JavaScript functions can be found there in un-obfuscated form. The sketcher monitors mouse movements, mouse button events (left and right button), and keyboard events for shortcuts. State involved with mouse operations is maintained on the client side in JavaScript variables. For example, a mouse-button-up event is reported to the server not just with the primary event and its drawing area coordinates, but also with the initial button-down coordinates, and the status of control keys. The server only stores the structure state, not all details about where and when the current event began.

Some critical events, such as mouse-up, are nearly always and immediately sent to the server - in case of mouse-up events the second event in a double click sequence is only discarded if the mouse coordinates did not change from the first click, and the first click is always reported. Others, like mouse movements, are extensively filtered. The number of original events generated by mouse movement is dependent on the browser and operating system, and in any case far exceeds the available bandwidth for image updating. The client-side JavaScript code, therefore, needs to throttle the sequence of events actually sent to the server for processing. A number of different techniques are applied for this purpose. First, a minimum distance of five pixels from the point of the last reported event is required. Since the server automatically snaps event coordinates to close objects, pixel-exact positioning is not needed. Second, the time since the last event report is taken into account. If this period is beyond a threshold of 333 ms and if the event would trigger a structure change or a change of graphical feedback elements, the event is transmitted to the server. Otherwise, a so-called "catch-up" event report is scheduled to be delivered in 200 ms. This timer is rescheduled anytime a new (dragged) mouse movement event is recorded. An approach such as this guarantees that an important event (and subsequently an image update) is reported every 333 ms if there is any major movement with display consequences. When the mouse movement stops, an event is also reported a maximum of 200 ms after the halt, if there is a need for a graphical update. This mechanism is used, for example, to make sure that, while drawing a bond, the bond line stays attached to the tip of the mouse cursor when the user slows down and navigates with precision. This also allows the bond line to lag during phases of rapid, less precise movement. In low-bandwidth mode, only catch-up mouse movement events are used, leading to a significant reduction of the number of events processed on the server. In typical editing operations, this leads to only ~30% of the number of mouse movement events being actually processed, as compared to the broadband setting. In combination with the suppression of anti-aliasing, the bandwidth requirement drops to about 20 Kbit/s.

Mouse events are not the only events associated with drawing area coordinates. Some keyboard shortcuts also use coordinates, such as ctrl-v for pasting a structure encoding from the clipboard onto the drawing area. Other keyboard shortcuts simply change the sketcher mode. A description of all keyboard shortcuts is available in the PubChem Sketcher help page http://pubchem.ncbi.nlm.nih.gov/sketch/sketchhelp.html.

The update of the status line is filtered by a 250 ms delay timer. Whenever the structure is likely to have changed, for example as the result of a mouse-up event on the drawing area and when the editor is not in certain purely graphical modes such as rotation or mirroring, a CGI request for the replacement of the contents of the hidden frame is scheduled. Again, this request is not sent but rescheduled if additional events occur. The timeout is, however, sufficiently short to guarantee that the status line contents have been updated at the time a user is able to, for example, perform a clipboard selection on the contents. When an update request is transmitted, the server sends back dynamically generated JavaScript which updates the status line and, potentially, other form fields of linked pages. Note that it is not possible to simply write two consecutive JavaScript requests for the update of both the drawing area and the status line. The structure information on the server-side is only updated when processing drawing area events. However, it is entirely possible and, in our experience, not uncommon that the status update request is received on the server before the image request because there is no guarantee of sequence preservation on independent network connections for those two requests. In that case, the status line would continue to report the conditions before the last image update.

It is noteworthy that the client-side JavaScript functions do not make use of any XMLHttpRequest object, the centerpiece of typical Web2.0 applications. The use of this construct does not yield any obvious benefits for this application, and our method does work on older browsers such as IE 4.0 which do not support this object.

## Server-Side Processing

The actual structure manipulations in response to events sent by the client are performed exclusively on the server side. In the beginning of a sketcher session, the main page of the sketcher is loaded by the client, where a session ID is transmitted to the server and either an empty structure object is created, a structure object is generated by decoding a linear notation string (e.g., a SMILES, SMARTS, or InChI), or a structure object is pulled directly from a PubChem database subsystem (e.g., by CID). This structure object, and a second backup object for undo/redo operations, remains associated with the session key.

When a client request is received, the structure object belonging to the client is identified via the session key, and manipulations are performed in accordance with the request. The response is either: an image of the updated structure, which is displayed in the sketcher drawing area on the client; an HTML page with embedded JavaScript commands sent to the invisible recipient frame, used to update the status line and other form elements; or, in the case of export commands, an actual chemical structure encoded in various graphical or chemical structure exchange formats. In any case, the resulting data is transmitted by the standard HTTP protocol, with a suitable MIME header prefixed to the actual result bytes.

Every server process can perform editing services for multiple parallel editing sessions. On a single-processor single-core server, a couple of dozen parallel sessions do not generate any load, since in most cases the computation time is only a few milliseconds, and, for a comparatively large fraction of the lifetime of a session, nothing really happens on the server other than interpretation of comparatively rare mouse events requiring action.

In case of a single server-side sketcher process, one easy approach to remembering the structure data is to simply keep it in memory. This is a mode well suited for sites without a lot of traffic (and indeed one of the state storage modes supported by the sketcher application). However, in the PubChem environment, with two load balanced multi-core, multi-processor servers each running four sketcher server processes, and where each server-side sketcher process can pick up an operation for any session at any time, a non-local structure state store is required. In that type of configuration, the structure data is retrieved from the store at the beginning of every task, via an unique key bound to the session ID, and, if anything is changed, written back into the same storage slot when the task has been completed. This is not as inefficient as it may sound. The content of the structure store is a simple serialized molecular data object, or more precisely a pair of these objects in order to support undo/redo functionality. Functions to encode and decode these objects efficiently are already part of the Cactvs toolkit. The data size of such an object, which stores basic connectivity plus atom and bond attributes, including query specifications, is usually a couple of kilobytes. The image data associated with a structure is not stored in these blobs since it needs to be regenerated after each structure change. The generic database blob storage mechanism is part of the PubChem queuing system. This system is implemented as monitored database tables on a redundant set of MS SQL Server hosts.

After considering the two sketcher extremes (single sketcher process on single server or many sketcher processes on many servers), there are two additional structure store mechanisms for an intermediate load configuration suitable for single-host multiprocessor systems. The structure data may be saved into shared memory segments or managed by a memory cache daemon (e.g., *memcached*[19]), where it can be picked up from multiple processes on the same server. The other option is to run the application script in a multi-threaded style, with a pool of threads picking up individual CGI tasks and threads operating concurrently on different processors. However, these two additional configurations are not currently deployed.

## Communication

Structure data from the sketcher is transferred to form input fields on pages which link to the sketcher by a push mechanism, not by invoking a function on the recipient form to retrieve the current sketcher contents. This push mechanism is part of the update scheme for the status line. Whenever the status line is updated, the server-generated JavaScript code which updates this form field will also attempt to call various functions with predefined names on the opener of the sketcher main window, if such a window exists. Arguments to these functions are the current structure content in various encodings. There is no real need for a specific "done" button on the sketcher mode panel. Its presence in the current interface is just due to user expectations. It simply closes the sketcher window and its operation is indistinguishable from clicking the equivalent window control element.

The function called in the secondary application window that opened the sketcher can fill the passed data into its application form fields or perform any other operation with it. For example, the sketcher is also used as a tool in a verification Web service for multi-record file uploads to be used in parallel multi-structure queries in PubChem. In this context, individual records of uploaded SD-files can be sent to the sketcher and edited. A thumbnail of that record is updated by the verification service with data received from the sketcher, by passing it to a structure rendering CGI. Since the transfer functions are called whenever the edited structure changes, the dependent form contents or other data representations are continuously updated during the editing process.

The classes of transfer functions, for which an attempt at calling is made, are configurable in the sketcher set-up. The underlying toolkit supports a large number of structure file formats, and any of these exchange formats can be used, as well as custom functions to transfer the molecular formula or various line notations such as SMILES, SMARTS, SLN, InChI, JME strings, PubChem Minimols (a compact file format designed specifically for PubChem structure searching) or CACTVS serialized objects. The transfer format used for PubChem structure queries is an extended, backwards-compatible SMARTS version.

## Usability and Usage Experiences

User feedback is generally very positive. In over two years, there is only a single reported instance of a user who could not get the sketcher to work because of severely restricted Internet zone control settings in their Web browser. Other observed failures were due to general server or network problems, not due to any specific unresolved issues with the sketcher implementation methodology. This may be surprising to some considering that approximately 15% of all PubChem Structure Search interactive users launch or otherwise consistently use the PubChem Sketcher on a daily basis.

We have set the various timers and timeout periods which control event processing to yield a maximum refresh rate of three to four images per second in broadband connection mode. The maximum updates rates are reached while performing drag operations with the mouse, where there is continuous feedback by trailing bond lines or the current location of moved or rotated structure parts. With these settings, reports from users in Europe and Japan indicated satisfactory usability even at these non-US locations. In regions with less developed infrastructure, it helps that the sketcher can be operated without resorting to any actions which provoke generation of a stream of feedback images. Almost all functions are accessible by means of isolated mouse clicks that change atoms, sprout bonds at default angles, change bond orders, add template fragments, and so on. If used in this style, the image is only updated when the structure has really changed, and there is no risk of an accumulating backlog of delayed image updates which are prone to confuse a user when they arrive late.

The sketcher has been used in unexpected and creative fashions by an enterprising user community. It has been praised as convenient interactive rendering tool for InChI and SMILES strings. We have also observed numerous attempts to access its rendering functions by batch scripts. Some of these access attempts had to be suppressed because they were not compatible with the NCBI access rules. In the sketcher configuration deployed on the PubChem site, the data transfer functions to fill linked forms are intentionally only usable only by pages served from within the NCBI domain. Linking the sketcher to other Web services with automatic data transfer is not supported.

## Summary

We have successfully implemented and deployed a new class of Web-based chemical structure editor. It has been in stable production use for more than two years. It is freely accessible at http://pubchem.ncbi.nlm.nih.gov/edit/.

By completely avoiding the need for a Java runtime, the installation of plug-ins and any advanced features in client Web browsers, we were able to provide an extremely portable chemical structure editing system which works on all major Web browsers, even those outdated by nearly a decade, and is entirely operating-system independent. The system has proven to be robust and reliable. The interactive responsiveness of the sketcher, while undoubtedly less than applications based on locally executed code in Plug-ins or Java virtual machines, was found to be sufficient for its purpose and we have received scant complaints. We therefore are convinced that our approach has demonstrated clear benefits over competing approaches for the input of chemical structures, especially in the context of public databases that have a diverse user base with a broad and unpredictable spectrum of browser software, operating systems, and Internet access methods.

## Availability and Requirements

The sketcher server application script and the complementary set of HTML pages and JavaScript functions are US government work in the public domain. They can be downloaded from ftp://ftp.ncbi.nlm.nih.gov/pubchem/CACTVS/. The server script can be run with a generic chemical Web script interpreter from the CACTVS toolkit (free for academic use, http://www.xemistry.com/academic) as long as no functions which require direct access to the PubChem databases (CID/SID/DID retrieval, queuing system database for state storage) are configured. The latter functions are not available outside NCBI, or, in case of CID and SID retrieval, only via different, slower conduits.

Installation instructions for the NCBI version of the sketcher are part of the manual, which can be downloaded as PDF or viewed as HTML pages. A working installation needs to be assembled from the Open Source portable application script and interface files downloadable from NCBI, which can be freely modified and redistributed, plus a proprietary generic closed-source script interpreter suitable for the target server platform, which is for example included in a feature-sufficient version in the standard academic distribution of the Cactvs toolkit and can be obtained from the Xemistry website.

The software is also available in an enhanced commercial version from Xemistry GmbH.

## Competing interests

EEB and SHB are NCBI employees. WDI is owner of Xemistry GmbH, and worked at NCBI as a contractor at the time of the initial sketcher implementation. Xemistry GmbH provides and supports more recent versions of the sketcher.

## Authors' contributions

WDI developed the core interpreter (existing software licensed to NCBI, thus not US Government Work), the application script and the user interface. EEB performed integration work. This paper was typed by WDI and EEB. SHB is the principal investigator for the PubChem project.
